# Phylogeny and antiquity of M macrohaplogroup inferred from complete mt DNA sequence of Indian specific lineages

**DOI:** 10.1186/1471-2148-5-26

**Published:** 2005-04-02

**Authors:** Revathi Rajkumar, Jheelam Banerjee, Hima Bindu Gunturi, R Trivedi, VK Kashyap

**Affiliations:** 1 National DNA Analysis Centre, Central Forensic Science Laboratory, 30 Gorachand Road, Kolkata- 70014, India

## Abstract

**Background:**

Analysis of human complete mitochondrial DNA sequences has largely contributed to resolve phylogenies and antiquity of different lineages belonging to the majorhaplogroups L, N and M (East-Asian lineages). In the absence of whole mtDNA sequence information of M lineages reported in India that exhibits highest diversity within the sub-continent, the present study was undertaken to provide a detailed analysis of this macrohaplogroup to precisely characterize and unravel the intricate phylogeny of the lineages and to establish the antiquity of M lineages in India.

**Results:**

The phylogenetic tree constructed from sequencing information of twenty-four whole mtDNA genome revealed novel substitutions in the previously defined M2a and M6 lineages. The most striking feature of this phylogenetic tree is the recognition of two new lineages, M30 and M31, distinguished by transitions at 12007 and 5319, respectively. M30 comprises of M18 and identifies a potential new sub-lineage possessing substitution at 16223 and 16300. It further branches into M30a sub-lineage, defined by 15431 and 195A substitution. The age of M30 lineage was estimated at 33,042 YBP, indicating a more recent expansion time than M2 (49,686 YBP). The M31 branch encompasses the M6 lineage along with the previously defined M3 and M4 lineages. Contradictory to earlier reports, the M5 lineage does not always include a 12477 substitution, and is more appropriately defined by a transversion at 10986A. The phylogenetic tree also identifies a potential new lineage in the M* branch with HVSI sequence as 16223,16325. Substitutions in M25 were in concordance with previous reports.

**Conclusion:**

This study describes five new basal mutations and recognizes two new lineages, M30 and M31 that substantially contribute to the present understanding of macrohaplogroup M. These two newly erected lineages include the previously independent lineages M18 and M6 as sub-lineages within them, respectively, suggesting that most mt DNA genomes might arise as limited offshoots of M trunk. Furthermore, this study supports the non existence of lineages such as M3 and M4 that are solely defined on the basis of fast mutating control region motifs and hence, establishes the importance of coding region markers for an accurate understanding of the phylogeny. The deep roots of M phylogeny clearly establish the antiquity of Indian lineages, especially M2, as compared to Ethiopian M1 lineage and hence, support an Asian origin of M majorhaplogroup.

## Background

During the last few years a surge in information on mitochondrial DNA control region sequence has been observed along with information from coding region substitutions in diverse populations of the world to understand their genetic diversity, structuring and origins [1-11]. More recently, mt DNA sequence data has been used to explore the peopling of Asia and to comprehend various population demographic parameters [12-20]. From a human genetics perspective India assumes importance in Asia because (1) the extensive diversity of populations residing in the sub-continent, which are biologically and culturally differentiated among themselves, (2) a clear distinction exists between non-tribal and tribal populations, autochthonous to the sub-continent [15,21], lastly and more importantly this expanse is believed to be one of the early regions of settlement of modern humans [20]. Of the four major matrilines identified till date L, M, N and R, about 60% of Indians trace their maternal roots in Indian specific branches of haplogroup M that is reported to have emerged from the African haplogroup L3. With the exception of the M1 lineage that is confined to Ethiopia [22], all the other branches of this macrohaplogroup including M*, C, D, G, E and Z haplogroups are observed in Asia [12,14,23,24]. The lineages M2, M3, M4, M5, M6, M18 and M25 are exclusive to India, with M2 reported to be the most ancient lineage in the sub-continent with an age estimation of 60,000 yrs-75,000 yrs. Furthermore, the frequencies of these clades among the different geographic, linguistic phyla and social strata have been investigated in detail, yet the fundamental question regarding origin of this super-haplogroup still remains unanswered [15,20]. While some authors have suggested a southwest Asian origin of M macrohaplogroup, followed by a back migration to Africa [15], others support its African ancestry [25]. One major drawback in arriving to a conclusion is the limitation of control region sequences in providing reliable estimate of phylogeny owing to homoplasy and recurrent mutations [23,26].

Complete mitochondrial genome sequencing has gained importance in resolving phylogenies and understanding human evolution. Extensive genome sequencing studies have been carried out in different lineages of L, N and M major- haplogroups across different global populations. Though the phylogenies of East Asian counter parts of M lineages: M7, M8a, M8C, M8Z, M9, E, D, G have been resolved in detail, but till date no similar studies have been attempted on the sub-lineages of the Indian M haplogroup [9,27-33]. The complete mt DNA sequence information from Indian M lineages will not only help answer questions regarding the origin and antiquity of this haplogroup, and resolving the phylogeny to finer branches but would also be highly relevant in understanding various parameters of population genetics, mitochondrial disorders and disease diagnosis and to some extent in forensic work [[28] and references therein].

The present study was undertaken to construct an unambiguous phylogeny for the M macrohaplogroup and to estimate its antiquity. Mitochondrial genomes were initially classified on the basis of their HVSI and coding region motifs, followed by complete sequencing of twenty-three samples representing different M matrilineals.

## Results

**Figure 1:**
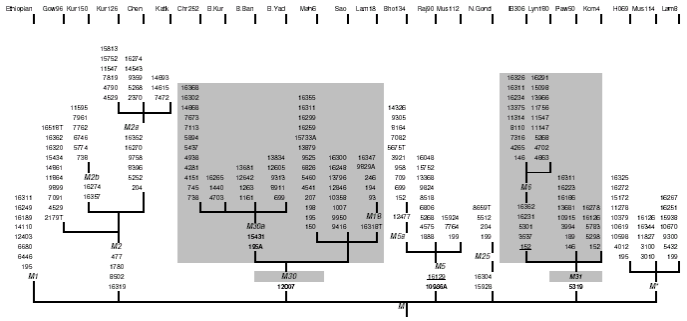
Phylogenetic tree of M macrohaplogroup based on complete mt DNA genome sequences. Numbers along links refer to substitutions at nucleotide positions with respect to rCRS. Suffixes are transversions. Grey colour represents potential new lineage. Asterisk denotes unclassified lineage. The M haplogroup differs from the rCRS at sites: 73, 263, 489, 750, 2706, 1438, 4769, 7028, 8701, 8860, 9540,10398, 10400, 10873, 11719, 12705, 14766, 14783, 15043, 15301, 15326, 16223.

We have found seven group defining basal substitutions and described fourteen lineages in detail from complete mt DNA genome sequence information, which would be helpful in further resolving some of the Indian M lineages. The M trunk differs from revised Cambridge reference sequence (rCRS) with substitutions at A73G, A263G, T489C, A750G, A2706G, A1438G, A4769G, C7028T, A8701G, A8860G, T9540C, A10398G, C10400T, T10873C, G11719A, C12705T, C14766T, T14783C, G15043A, G15301A, A15326G and C16223T. The coding region mutation sites analyzed in the present study were different from those observed in the sister M1 lineage found in Ethiopia. The M phylogenetic tree constructed on whole mt sequence information of twenty four samples belonging to different M lineages and their sub-types including M1, M2, M2a, M2b, M30, M30a, M18, M*, M3, M4, M5, M6, and M25 is provided in Figure 1.

### M2 lineage

The complete sequencing of five mt DNA genomes belonging to M2 and its sub-lineages, M2a and M2b, indicated that coding region mutations T477C, T1780C, A8502G were associated with HVSI motifs C16223T and G16319A, which formed the root of M2 lineages. In case of M2a, we report a novel basal substitution at site T9758C in addition to previously reported transitions at G5252A and A8396G. Screening of T9758C site in twenty seven Indian individuals possessing M2a specific HVSI and coding region motifs, clearly establishes this mutation as a marker of this sub-lineage. The M2b sub-lineage having HVSI motif G16274A and T16357C, in addition to M2 defining mutations sites did not share any coding region substitutions with M2a. Overall, M2 lineage presents the maximum number of coding region mutation sites than any other analyzed lineage in this study.

### M30 lineage

A new lineage M30 was recognized in the M macrohaplogroup, comprising of seven mt genomes, six of whose HVSI motifs did not correspond to any of the earlier established M lineages and one that was recently identified as M18 lineage (represented as shaded region in Fig 1). Since lineages of M have already been catalogued from M1 to M25, this potential new lineage is designated as M30 to avoid any ambiguity in classification of M macrohaplogroup. This branch arises from the main M trunk with transition at site G12007A. Finer resolution of this lineage was achieved by further clustering four complete sequences with mutation at two sites, T195A and G15431A into a sub-lineage designated as M30a. A total of eighteen Indian individuals belonged to M30a lineage. Three mt DNA genomes further branched out from M30 lineage, possessing only G12007A substitution. Interestingly, the newly described M18 (C16223, A16318T) matriline is one of the branches that directly emerged from M30 lineage. Sequence analysis of ten M18 mt DNA genomes showed the presence of G12007A transition. Eighteen Indian individuals were identified from our mtDNA database as possessing a HVSI motif (C16223T, A16300G) similar to the Saora sample, which arose as an offshoot of M30 lineage. All eighteen individuals tested positive for G12007A transition, suggesting that it might be acceptable to place M18 under M30 lineage.

### M5 lineage

The basal motif T12477C, G16129A and C16223T describes M5 lineage of majorhaplogroup M. Whole genome sequencing of three samples with similar HVSI motif, G16129A and C16223T, revealed that only one sample (I. B306) exhibited the T12477C mutation, and designated as M5a sub-lineage (Fig 1). This site was nevertheless absent in the other two samples, one of which had a similar HVSI motif as M5a mt DNA genome, while the other exhibited an additional site G16048A in its control region motif. Our study identifies a transversion at C10986A, shared by all the three samples, suggesting that these sequence types branched out from a common root. Analysis of C10986A substitution in seven Indian samples possessing HVSI motif, G16048A, G16129A and C16223T, confirms our finding that different branches emerged from M5 lineage. It is, however, important to note that G16129A might not be important in defining M5 and C10986A marker remains confined to this lineage until tested in a sufficiently large number of undefined M*samples.

### M25 lineage

The M25 lineage has been recently described by the presence of G15928A and T16304C. It differs from the M halogroup by five coding region substitutions and arises directly from the M trunk with no additional group defining motifs.

### M31 lineage

The present analysis defines another new lineage that emerged from the main trunk of M macrohaplogroup, with a substitution at A5319G. Keeping in succession with the numbering of lineages, we designate this lineage as M31. This branch comprises of the previously well-defined M6 lineage along with M3 and M4 lineages.

The two M6 matrilines completely sequenced, harbor the characteristic group defining mutations at site T16231C, T16362C and C3539T. Our analysis deciphered another novel substitution at site A5301G in this lineage.

Whole sequencing of two M3 genomes (defined by T16126C transition) demonstrated a lack of similarity in their coding region substitutions. Since both T16126C and C16311T are fast mutating sites, we propose the non-existence of M3 and M4 lineages in the M phylogeny.

Three mt DNA genomes could not be differentiated in the study and were left designated as M*. One of the M* genomes was previously characterized as M3 lineage and differed in its coding region substitutions from the other M3, currently placed under M31 lineage. The matrilineal type possessing C16223T and T16325C as HVSI motif has been observed in relatively high frequency in Indian populations. Contrary to our expectation, full sequencing of this mt DNA (Ho69), did not exhibit the presence of G12007A mutation site that was observed in other unidentified M lineages analyzed in this study. Similar results were observed after complete sequencing of the HVSI motif type C16223T and C16251T.

The newly described phylogeny based on twenty-three Indian specific mt DNA sequence information radiates from the trunk of M macrohaplogroup with limited number of branches, differing in their basal motifs with one or more coding region substitutions. The branching pattern displayed by M2, M30, M31 and their sub-lineages mirrors the expansion model, indicating that most of the lineages described on basis of their control region sequence, occupy peripheral positions of the phylogeny. M2 is estimated to be the oldest lineage with an age of 49,686+/- 10,903 years before present (YBP). Furthermore, sub-lineage M2a differs from M2 by a minimum of six coding region substitutions, reiterating the antiquity of M2. The newly defined M30 and M31 lineages differ from the root of M by six to eight substitutions and hence, display similar expansion ages of approximately 33,042+/- 7,840 YBP.

## Discussion

Analysis of short stretches of mt DNA HVSI and HVSII region have significantly aided in clearly discriminating some of the M lineages. With the aim of understanding migration routes of diverse Indian people, more control region sequences are being generated without much support from coding region sites, resulting in an increasing number of conflicts within the classification of its lineages. We report a phylogenetic tree constructed from the whole genome sequencing of twenty-three Indian and one Ethiopian M lineage to resolve some of the anomalies occurring due to recurrent mutations in control region.

The control region sequences have exhibited the presence of an array of M lineages in India [12,16,20], despite which, complete mt DNA sequencing suggests that most of these lineages arose as limited offshoots of the main M trunk. The newly constructed M phylogeny displays difference in the branching patterns of lineages, with total number of substitution sites varying even within a lineage. Substantial variation in branch length within a lineage is indicative of the existence of further branching that could probably be delineated with generation of additional data. The M2 genome and its sub-lineages, more specifically M2a, have been well described in Indian population. Nevertheless complete sequencing of M2a demonstrated the presence of a novel site T9758C, which is characterized as a diagnostic marker for M2a sub-lineage, in addition to the previous reported, G5252A and A8396G transitions [15]. This finding reinforces the importance of sequencing a large number of individuals belonging to a lineage for describing a detailed phylogeny. The study was unable to trace any specific marker for M2b sub-lineage. In the absence of any lineage specific marker for M2b till date, it might be suggested that this sub-lineage is not a distinct clade of M2 lineage but is an M2 with additional HVSI motif (G16274A, T16357C). However, more M2b genomes need to be completely sequenced before reaching this conclusion and though HVSI sequences are not very reliable for constructing phylogenies, this cluster can well differentiate individuals with only one or both mutations and in turn resolve the phylogeny to its finer sub-lineages. M2 lineage is the oldest M lineage found in India with an estimated age of approximately 50,000 YBP, using only coding region motifs estimation, opposed to the expansion date of 60,000–75,000 yrs calculated from control region sequence information [15].

Although the G12007A substitution has been previously identified in other haplogroups, besides the M lineages [29], this study presents a novel lineage M30 that was differentiated to include mitochondrial genomes possessing G12007A substitution. The erection of M30a sub-lineage with its root at T195A and G15431A may help in further classifying M* samples that have yet to be identified owing to the absence of any characteristic HVSI motif.

Mitochondrial genomes possessing the 16223, 16300 motif appear to be a promising new sub-lineage arising from M30. Additional complete mtDNA sequencing of similar sub-types may further help in precisely defining this branch. The M30 lineage was relatively younger than the M2 lineage with an expansion age of approximately 33,000 YBP, calculated on the basis of its coding region sequence information.

An important contribution of this study is placement of M18, M6 and previously defined M3 and M4 lineages in the M phylogeny. In the absence of a coding region marker for M18 lineage [20], G12007A substitution provides a stable root to M18 type, which is defined only on basis of the HVSI motif A16318T. The recognition of M31 lineage with an A5319G basal transition further reduces the number of branches arising from the trunk of M lineage. Since M6 is already well characterized, we propose that it remain as a sub-lineage of M31. However, it is essential that previously defined M3 and M4 lineages be completely removed from the phylogeny. Furthermore, it might be realized that the newly defined M4a lineage [20], might in fact be a sub-lineage or independent lineage by itself.

The M phylogenetic tree has largely aided in resolving the position of M5 lineage. Until recently, transition at G16129A along with basal motif of M, was used to characterize this lineage [34] and is currently described by the presence of coding region mutation at T12477C [20]. The phylogenetic tree constructed on the basis of complete mt DNA genome sequencing provides evidence to support our finding that at least two sub-lineages arise from M5 that share a transversion at site C10986A and may or may not possess T12477C transition. Presence of T12477C transition in only one of the two M5 mt DNA genomes sharing an identical HVSI motif, C16223T and G16129A, further substantiates the importance of coding region markers in precisely identifying mitochondrial phylogenies. Even though G16048A, HVSI motif has not been included under M5 owing to absence of T12477C, this study includes this motif under M5 lineage. However, prior to defining G16048A, G16129A and C16223T cluster, it is imperative that more samples representing this HVSI motif be completely sequenced. The age of M5 lineage is estimated to be 34,095+/- 6,425 YBP, indicating that M5 and its sister lineages M30 and M31 probably branched out from M haplogroup around the same time.

The newly defined M25 lineage did not share mutation sites with any other lineage and independently arose from M trunk with G15928A and T16304C substitutions.

The moderately high frequency of C16223T, T16325C HVSI motif types in Indian samples suggests that there might be a potential new lineage, which might be more accurately described once additional genomes possessing this motif are fully sequenced. In the absence of this sequence information, no attempt was made to classify this sequence type in to a lineage and hence, designated as M*. The other M* lineages bearing the control region motif, C16223T, T16126C and C16223T, C16251T, C16267T could not be resolved further for similar reasons.

Although, this study presents only a preliminary view of the M phylogeny, yet the emerging data may be highly useful in resolving the long-standing debate on Asian origin of M macrohaplogroup. Since M macrohaplogroup is derived from L3, which finds its roots in East Africa, it is believed that presence of M1 in Ethiopia further substantiates an African origin of M. A similar hypothesis had been drawn for the U6 lineage that is autochthonous to North Africa although U haplogroup displays its maximum diversity in Near East [35]. The authors of this study prepared an in-depth phylogeography of U6 to infer a back migration of this lineage from West Asia to North Africa. In the absence of a detailed M1 phylogeny, we have focused our attention on M2 to estimate the place of split of M from L3 as Africa or Asia. Interestingly, a single M2 genome differs in its coding region from the root of M at ten sites as compared to M1, which possess only four substitutions. Also, sub-lineages of M2, M5, M30 and M31 show long branch lengths, highlighting the deep roots of these lineages. Considering the antiquity of M2 and other East Asian specific M lineages [33], Ethiopian M1 lineage is by far a relatively newer branch. Our study on M1 and M2 mitochondrial genomes clearly established the Asian origin of M macrohaplogroup, followed by a back migration to Africa. We further suggest that as more M1 mt DNA genomes are sequenced, there is a possibility that this lineage might find its root in one of the peripheral branches of Asian M lineage.

## Conclusion

This study presents the, first ever, phylogenetic tree constructed for the M lineages predominant in India. The phylogenetic tree emerged from the present study consists of seven branches. A significant achievement of this endeavor is the recognition of M30 and M31 lineages, which encompass seven and three sub-lineages respectively. While the previously defined lineages, M18 and M6 are now found to be sub-lineages of M30 and M31; our study suggests the non-existence of M3 and M4 as independent lineages. Thus, the present analysis also determines the roots of few previously undefined mt DNA genomes, reinforcing the importance of screening lineage defining coding region substitutions before describing a lineage. Another significant conclusion emerged from this study is substantiating the Asian origin hypothesis of M macrohaplogroup. This study would provide baseline information for precisely describing the macrohaplogroup M, as more complete mt DNA sequence information is generated. Furthermore, it establishes the superfluousness of control region diversity reported in the M macrohaplogroup by suggesting that most of M lineages might in fact be derived from limited basal branches.

## Methods

### Amplification and sequencing of HVSI and coding region motifs

Genomic DNA was extracted from whole blood by standard Phenol/chloroform method. Amplification and sequencing of control region were performed in 1258 samples (authors unpublished data) as described in our earlier studies [36,37]. Samples were initially analyzed for substitutions at site 10397 and 10400 via RFLP protocol before characterizing them under M haplogroup and subsequently clustered into different lineages of M via sequencing of the required coding region fragments- T447C, T1780C and A8502G for M2, G5252A for M2a, T12477C for M5, C3539T for M6 and G15928A for M25 [15,20].

### Complete mtDNA sequencing

Socio-cultural affiliations of twenty-three individuals selected for complete sequencing are presented in Table 1. These mt DNA genomes belonging to M* (n-8), M2 (n-1), M2a (n-3), M2b (n-1), M3 (n-2), M4 (n-1), M5 (n-3), M6 (n-2), M18 (n-1) and M25 (n-1) sub-lineages of Indian macrohaplogroup M were completely sequenced in the current study. DNA amplification and sequencing was carried out using primers described elsewhere [38]. New group defining substitutions were re-sequenced, and their frequency was determined in other Indian samples with similar control region motifs. Each sample was completely sequenced twice to remove any ambiguous sites. Additionally, since several segments of the same mt DNA had to be screened, care was taken to avoid artificial recombination caused by potential crossovers.

**Table 1:** Detailed population information of 23 mitochondrial genomes analyzed in current study.

S.No.	Sample ID	Population	Social status	Ethnicity	Geographical origin	Linguistic Affiliation
1	Gow96	Gowda	Caste	Australoid	Karnataka	Dravid
2	Kur150	Kuruva	Tribe	Australoid	Karnataka	Dravid
3	Kur126	Kuruva	Tribe	Australoid	Karnataka	Dravid
4	Chr252	Christian	Caste	Australoid	Karnataka	Dravid
5	Bho134	Bhovi	Caste	Australoid	Karnataka	Dravid
6	Mus112	Muslim	Caste	Australoid	Karnataka	Dravid
7	Mus114	Muslim	Caste	Australoid	Karnataka	Dravid
8	IB306	Iyengar Brahmin	Caste	Caucasoid	Karnataka	Dravid
9	Lyn180	Lyngayat	Caste	Caucasoid	Karnataka	Dravid
10	Chen	Chenchu	Tribe	Australoid	Andhra Pradesh	Dravid
11	Lam18	Lambadi	Tribe	Caucasoid	Andhra Pradesh	Indo-European
12	Lam8	Lambadi	Tribe	Caucasoid	Andhra Pradesh	Indo-European
13	N.Gond	Naikpod Gond	Tribe	Australoid	Andhra Pradesh	Dravid
14	Kom4	Komati	Caste	Australoid	Andhra Pradesh	Dravid
15	B.Kur	Kurmi	Caste	Caucasoid	Bihar	Indo-European
16	B.Ban	Baniya	Caste	Caucasoid	Bihar	Indo-European
17	B.Yad	Yadav	Caste	Caucasoid	Bihar	Indo-European
18	Raj90	Rajput	Caste	Caucasoid	Bihar	Indo-European
19	Katk	Katkari	Tribe	Australoid	Maharashtra	Indo-European
20	Paw50	Pawara	Tribe	Australoid	Maharashtra	Indo-European
21	Sao	Saora	Tribe	Australoid	Orissa	Austro-Asiatic
22	Mah6	Mahali	Tribe	Australoid	West Bengal	Austro-Asiatic
23	Ho69	Ho	Tribe	Australoid	Jharkhand	Austro-Asiatic

### Phylogenetic analysis

The sequence information generated by whole mt genome sequencing of twenty Indian specific M lineages was used to construct a phylogenetic tree of macrohaplogroup M. Substitutions were reported with respect to the revised Cambridge Reference Sequence (rCRS) [39]. Only those mutation sites occurring in all sequenced genomes of a maternal lineage were placed at the root. The complete sequence of the Ethiopian M1 genome [25] was included in the M Phylogenetic tree to determine its relationship with its sister lineages.

### Time estimate

Age estimates for the M lineages were computed using only coding region substitutions identified from the complete mt DNA genome sequencing. The mean number of mutations per site to the most recent common ancestor was estimated and converted to real time using a substitution rate of 1.26 × 10^-8 ^per site per year [40].

## Authors' contributions

RR, JB and HB carried out carried out extensive sequencing, RFLP experiments and also analyzed the genetic data. RR did phylogenetic analysis and drafted the manuscript. RT provided critical and valuable information during processing of data. VKK is responsible for conceiving and designing of the study and contributed significantly in interpretation of data and shaping of the manuscript. All authors read and approved the final manuscript.

## Electronic reference

Rajkumar R, Banerjee J, Gunturi HB, Trivedi R, Kashyap VK: Phylogeny of the M superhaplogroup inferred from complete mitochondrial genome sequence of Indian specific lineages. Genome Biology 2004, 6:P3 
